# Bull Dogs

**Published:** 1859-04

**Authors:** 


### HALL'S JOURNAL OF HEALTH.

OUR LEGITIMATE SCOPE IS ALMOST BOUNDLESS: FOR WHATEVER BEGETS PLEASURABLE AND HARMLESS FEELINGS, PROMOTES HEALTH; AND WHATEVER INDUCES DISAGREEABLE SENSATIONS, ENGENDERS DISEASE.

*We aim to show how Disease may be avoided, and that it is best, when sickness comes, to take no Medicine without consulting an educated Physician.*

VOL. VI.] APRIL, 1859. [No. 4.

### BULL DOGS.

When quite a child, a beautiful big dog came to our father's house, no one knew whose or whence. All the children were wonderfully taken with him; he was fed and caressed and played with, from morning till night, and we all thought we had gotten a valuable prize. Before long, however, we discovered a failing, a serious draw back; there was no reliability in his mood; for in the very midst of our gambols with him, he would sometimes turn round and snap at us so savagely, that we began to avoid him. Strangers would often exclaim "what a beautiful dog you have!" But we could not join in any commendation of him. We let visitors praise him, and we let him alone.

Later in life, we have found bull dogs everywhere, in every party, in every sect, in every profession, and in very many families.

A young man is a suitor, his dress and address mark the gentleman. He is educated, travelled, handsome. His demeanour is unexceptionable, and he wins the hand and trusting heart and makes them his own. But on a nearer view, after marriage, unexpected developments are made, startling principles are enunciated, the principles of the *roue*, of the gambler, of the infidel; with such an one a pure heart can never assimilate, and retires more and more within itself, while the other left more and more to itself, grows cold and fretful; becomes daily more soured, and complaints, and faultfindings, and growls, are the order of the day—that is a Domestic Bull Dog.

A strange physician arrives, he is polished in his manners, plausible in his theories, and confident in himself. Courteous in deportment, agreeable and gossipping in conversation, he wins his way among the people; they forsake the man to whom they have been bound by ties of citizenship and near neighborhood for a dozen or twenty years, and the new comer is all and all. But time developes character. With a remorseless maw, he snaps at his new patrons' purses, bites out in merciless mouthfuls the substance of his patients, who just about that time find out that he is not as good as their "old doctor." But the new one got their purse, and they got their experience by paying the—Medical Bull Dog.

A minister comes among us, we never heard of him before, but he "walks into our affections" unresistingly, for we are carried away with his eloquence. As lavishly as corn grains to a brood of chickens, does he scatter around him the bright jewels of thought; we feel as if we could sit and listen to him always, and he settles among us. But no sooner fixed, than some idea is proposed, which we do not like altogether, but thinking that we must have heard amiss it is passed over, and for "a spell," all moves on smoothly as before: then another new idea is thrown out, rather more rousing than before, in fact it is disquieting; and with the charity which many good qualities engendered, we think perhaps he did not mean what he said, had failed to express himself clearly; but before the irritation has subsided, another shot is cast, and another and another, with shortening intervals, until not a sermon is heard without some expression is made more or less startling, enough to make us feel that it is nothing short of a desecration of the day and the place and the occasion. These things go on until by degrees the new-comer is "shied" from by the more reflecting; they cease to wait on his ministrations, say nothing in his praise, and let him alone. Next the newspapers take him up, they handle him gingerly at first, but his sentiments, and his conduct becoming more and more "liberal" in an ungracious sense, he is, after much long suffering, in consequence of his undenied mental power and other bright qualities, reluctantly "read out," and he settles down among the heterodox and the infidel, where he belonged from the first, and thenceforward is regarded as a Clerical Bull Dog.

A daily, a weekly, a monthly, a quarterly publication is left at our doors. A close criticism discovers nothing objectionable and much to commend. It comes too, at a low price, and we conclude to give it the support of our patronage and influence. It continues good, and by degrees we begin to feel a personal interest in its prosperity; and about this time, the rise in price to that of others of its class, is announced, we wince and bear it. Later still, there is a latitudianarism in its editorials, not wholly agreeable; these gradually grow more and more decided, to become in time as dogmatical, as impertinent, as levelling as any of its class, and we tolerate when we do not admire; and as we can't better ourselves, we submit, to be aroused to indignation even, at sentiments uttered every now and then, political, social, religious, which almost determine us not to take that paper another day. But we must have a paper, it is no worse than the others, while in some things it is better, and we take it still, forgetting that an arrow poisoned with a false doctrine in politics, in domesticities, in religion, especially when barbed with ridicule, never fails to leave in young minds a venom which remains and rankles and corrupts to the utter ruin sometimes of the whole moral character.— Beware then of Editorial Bull Dogs.

The dog which came to our father's house had no doubt been kicked out of somebody else's: we at length did the same thing, and he slunk off to find another home. He was a peripatetic bull dog, his prototype is found in those who go about the country lecturing professionally on this, that, or the other specified subject; but to cut the whole matter short, we will state it as our observation, that with very few exceptions, we come away from a public lecture with feelings varying from dissatisfaction to disgust, and now and then with horror; for no later than last night, having for the reason above given, almost wholly ceased from attending public lectures, we heard a man discoursing professedly on "Fun;" we love a laugh, for we know it to be a better pill for the dispersion of blues, inanity and the like, than any of our compounding, hence we go willingly where a whole-souled risibility may be reasonably expected. The lecturer pleased us hugely at first. He hit off gaming, and profanity, and drunkenness to a T, closing, however, with the laudification of *Punch*, and Thackeray, and Dickens, making quotations from these men, as being superior to any sentiment from any pulpit in Christendom, and with a twitting of parsons and of people, who were so pious that a smile was considered a profanity, he ceased with the growl of a Bull Dog Lecturer. The lesson of the article is—beware of new men, of strangers. Take time to "try the spirits." Of social bull dogs, domestic bull dogs, and bull dogs medical, as also those of the press, the rostrum and the pulpit, beware!

